# Alumni Association

**Published:** 1869-05

**Authors:** S. A. McWilliams

**Affiliations:** Secretary and Treasurer, 166 State St.


### ALUMNI ASSOCIATION.

Minutes of the annual meeting of the Alumni Association of Chicago Medical College:—

The members of the Alumni Association of Chicago Medical College met in the lower lecture room of the College at 7½ P.M., March 22, 1869.

The President, Dr. H. P. Merriman, occupied the chair.

The attendance was larger than expected.

Minutes of previous meeting read and approved.

Some amendments were made to the Constitution and By-Laws.

Interesting papers were read from Drs. Herbert Harris, D. A. Sheffield, J. F. Kelsey, A. C. Corr, G. Wheeler Jones, Stacy Hemmenway, and others.

The President's address was then listened to with deep interest by all; a copy of which, on motion, was requested to be furnished for publication in the *Chicago Medical Examiner*.

The following are the officers elected for the ensuing year:—

*President*, J. S. SHERMAN, M.D., Chicago.

*1st Vice-President*, J. G. FREDIGHE, M.D., Chicago.

*2d Vice-President*, G. H. FULLER, M.D., class '69.

*Secretary and Treasurer*, S. A. MCWILLIAMS, M.D., Chicago.

The Alumni were then notified of the Treasurer's willingness to receive their annual dues.

No farther business appearing, the meeting adjourned.

At a subsequent meeting, the Association voted fifty dollars for a prize to that member of the Association who should present the best essay on some medical or surgical topic. Dr. N. S. Davis offers to increase the sum to one hundred dollars. The subject of the essay to be left to the option of the competitor. All are members who have paid their yearly dues of one dollar. Any Alumnus not now a member can become one by sending his name and one dollar to the Secretary, which it is hoped every one will do.

The Committee on competing essays consist of N. S. Davis, M.D.; E. Andrews, M.D.; H. A. Johnson, M.D.; and W. Hay, M.D. Each essay must be designated by a device or motto, and must be accompanied by a sealed envelope bearing the same device or motto, and containing the name and address of the author. The envelope belonging to the successful essay will be opened and the name of the author announced at the next annual meeting of the Association, also, at the next annual commencement of the College in 1870. The competing essays must be sent in to the Secretary of the Association on or before the 15th February, 1870. It is expected that the prize essay and others of marked value will be published in pamphlet form and furnished to the members of the Association.

S. A. McWILLIAMS, *Secretary and Treasurer,*
*166 State St.*

